# Primate malarias as a model for cross-species parasite transmission

**DOI:** 10.7554/eLife.69628

**Published:** 2022-01-28

**Authors:** Marina Voinson, Charles L Nunn, Amy Goldberg

**Affiliations:** 1 Department of Evolutionary Anthropology, Duke University Durham United States; 2 Duke Global Health, Duke University Durham United States; Johns Hopkins Bloomberg School of Public Health United States; Pennsylvania State University United States

**Keywords:** primate malaria, host switching, host-parasite interactions, *Plasmodium*, zoonosis

## Abstract

Parasites regularly switch into new host species, representing a disease burden and conservation risk to the hosts. The distribution of these parasites also gives insight into characteristics of ecological networks and genetic mechanisms of host-parasite interactions. Some parasites are shared across many species, whereas others tend to be restricted to hosts from a single species. Understanding the mechanisms producing this distribution of host specificity can enable more effective interventions and potentially identify genetic targets for vaccines or therapies. As ecological connections between human and local animal populations increase, the risk to human and wildlife health from novel parasites also increases. Which of these parasites will fizzle out and which have the potential to become widespread in humans? We consider the case of primate malarias, caused by *Plasmodium* parasites, to investigate the interacting ecological and evolutionary mechanisms that put human and nonhuman primates at risk for infection. *Plasmodium* host switching from nonhuman primates to humans led to ancient introductions of the most common malaria-causing agents in humans today, and new parasite switching is a growing threat, especially in Asia and South America. Based on a wild host-*Plasmodium* occurrence database, we highlight geographic areas of concern and potential areas to target further sampling. We also discuss methodological developments that will facilitate clinical and field-based interventions to improve human and wildlife health based on this eco-evolutionary perspective.

## 1. Introduction

Animals host an incredible diversity of parasites, here defined as organisms that live in or on another organism (the host) at some cost to the host, including microparasites (viruses, bacteria, fungi, and protozoa) and macroparasites (helminths and arthropods). Science is only just starting to understand this diversity of parasites, with the vast majority of parasites yet to be documented (Dobson et al., 2008; Poulin et al., 2016; Carlson et al., 2019). Some parasites are highly host specific, meaning that they are found only on a single host species, while others are generalists that are able to infect multiple hosts. Hence, these symbiotic associations and their transmission represent a vast web of connections that can be mapped among host species (Poulin, 2010; Gómez et al., 2013). These associations vary over time as parasites go extinct, speciate, and transmit across host species, with these processes influenced by evolutionary dynamics and geographic movements of the host species themselves (Combes, 2001). Parasites also drive coevolutionary dynamics involving reciprocal selective pressures favoring host defenses and parasite adaptations to overcome those defenses.

The factors that drive the connections between hosts and parasites are central to major research programs in ecology and evolution. These associations, and changes to them, also impact human health. In particular, parasites and pathogens can shift to human populations (a zoonosis) and adapt to humans, in some cases evolving to become specialists on humans (Wolfe et al., 2007), as seen with HIV-AIDS, measles virus, and the malaria parasite *Plasmodium falciparum*. Given the massive and global extent of anthropogenic change and its impacts on disease-carrying hosts (Gibb et al., 2020), such events are likely to occur increasingly often. Cross-species transmission events are also important to animal health and conservation, with parasites having negative fitness consequences for animal hosts and contributing to extinctions (De Castro and Bolker, 2004). Many of these negative outcomes result from cross-species transmissions from domesticated animals, invasive species, or humans (known as anthropozoonoses in the latter case). Finally, the loss of a host causes loss of parasites (Koh et al., 2004; Dobson et al., 2008; Dunn et al., 2009; Herrera et al., 2021). Given the important role of parasites in ecosystems, the loss of hosts can have cascading effects, with some authors proposing using naturally occurring parasites as a marker of a healthy ecosystem (Hudson et al., 2006).

A phenomenon of particular importance for global health is parasite sharing, which refers to the occurrence of a parasite in multiple host species. The distribution of parasites across hosts is influenced by three mechanisms. The first of these is co-speciation, with the diversification of the host resulting in diversification of the parasite. This scenario results in congruent host and parasite phylogenies, as found in primates and their pinworms (Hugot, 1999). Co-speciation is expected to result in parasites specializing on particular hosts (or sets of closely related hosts). A second mechanism involves opportunistic transmissions from one host species to a new species, broadly known as a host shift. Once successfully infecting a new host, the parasite lineage may specialize on it. Finally, a generalist parasite may infect multiple hosts. The majority of parasites may fall into this category, with sharing either limited to a few hosts – as is the case of Ebola virus infecting bats, great apes, and duikers – or to a wide range of hosts – as in the case of *Giardia* infecting many phylogenetically diverse species. In primates, for example, one study found that approximately 70% of known parasites are documented to infect more than one host (Pedersen et al., 2005).

Here, we review parasite sharing between humans and our close primate relatives for a group of protozoan parasites that cause malaria. We discuss how evolutionary and ecological perspectives can inform the origin and virulence of emerging zoonoses, as well as pathways for vaccine or therapeutic targets. Malaria parasites range from single-host specialists to wide generalists, with different malaria parasites infecting a broad range of animals, including birds, bats, primates, lizards, ungulates, and rodents (Galen et al., 2018). Host sharing of malaria parasites is driven by a mix of ecological and genetic factors, and our understanding of the process is biased by sampling of some hosts more than others (Garamszegi, 2009; Faust and Dobson, 2015). For example, among avian malaria species, previous studies revealed that malaria species tend to be generalists that infect a wide range of host species, allowing them to invade new ecosystems (Ricklefs and Fallon, 2002; Gupta et al., 2019; Ewen et al., 2012; Galen et al., 2018), though the full diversity and ecology of these species is only starting to be appreciated.

Human-infecting malaria parasites are part of the genus *Plasmodium* (Galen et al., 2018; Sharp et al., 2020). These protozoan parasites have an obligate *Anopheles* mosquito vector stage for sexual reproduction and transmission between hosts. Of the roughly 30 known primate malaria parasites, currently a handful are known to naturally infect humans regularly: *P. falciparum*, *Plasmodium vivax, Plasmodium malariae, Plasmodium ovale wallikeri,* and *Plasmodium ovale curtisi,* with growing evidence that *Plasmodium knowlesi* is also a natural parasite of humans (Singh et al., 2004; Sharp et al., 2020). Indeed, all human malaria parasites have a zoonotic origin from our nonhuman primate (NHP) relatives. Ancient host switching includes two of the most common human malaria parasites, *P. falciparum* and *P. vivax*, which are now endemic in humans, and rare in NHPs. Ongoing and emerging zoonoses include *P. knowlesi*, *Plasmodium simium,* and perhaps *Plasmodium brasilianum* (Antinori et al., 2021; Faust and Dobson, 2015).

**Figure 1.**
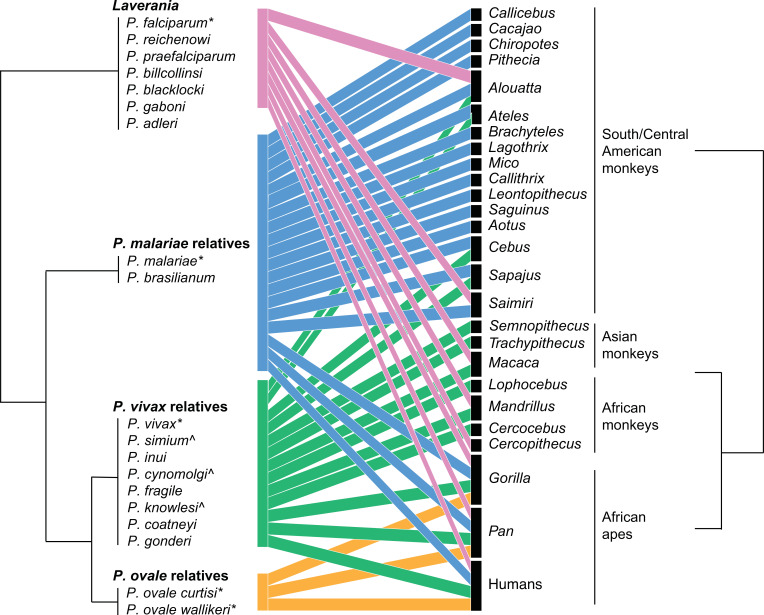
Bipartite plot of malaria parasite clade infection in primate genera. Phylogeny of malaria parasites follows Sharp et al., 2020, with parasites grouped by their clade based on the primary human-infecting parasite in that clade; clade names used in the main text are above parasite groups. Branch lengths are arbitrary. Colors correspond to the parasite clade. Figure made in R bipartite package (Dormann et al., 2008). * denotes common human-to-human-transmitting parasites, and ^ denotes nonhuman primate (NHP) parasites that have been found to naturally infect humans.

Figure 1 summarizes the host-parasite relationships between the major clades of primate malaria parasites and the primates that they infect. The lack of high-quality whole-genome data for some primate malarias makes the phylogenetic relationships between certain parasites unclear and open to change (Pacheco et al., 2013; Arisue et al., 2019; Galen et al., 2018; Loy et al., 2018; Daron et al., 2020). Notably, *Plasmodium* is a paraphyletic genus name; *P. vivax* is more closely related to rodent malarias, such as *Plasmodium berghei* and *Plasmodium chabaudi*, and to *Hepatocystis* spp., than it is to *P. falciparum* (Galen et al., 2018; Sharp et al., 2020). Hence, the historical naming of *Plasmodium* should be supported by more taxonomically consistent subgenic taxonomic definitions based on the main clades (Figure 1). Therefore, we focus on this higher taxonomic level. The subgenus naming convention we use, along with the species within each subgenus, is listed in Figure 1. When referring to single *Plasmodium* species, we will use the species name, for example, *P. vivax*; when referring to the broader clade, we will use the term ‘relatives,’ for example, *P. vivax* relatives or *P. vivax*-related, except for *Laverania*, which is already widely used to describe the subgenus including *P. falciparum* and other parasites that infect great apes.

**Figure 1—figure supplement 1.**
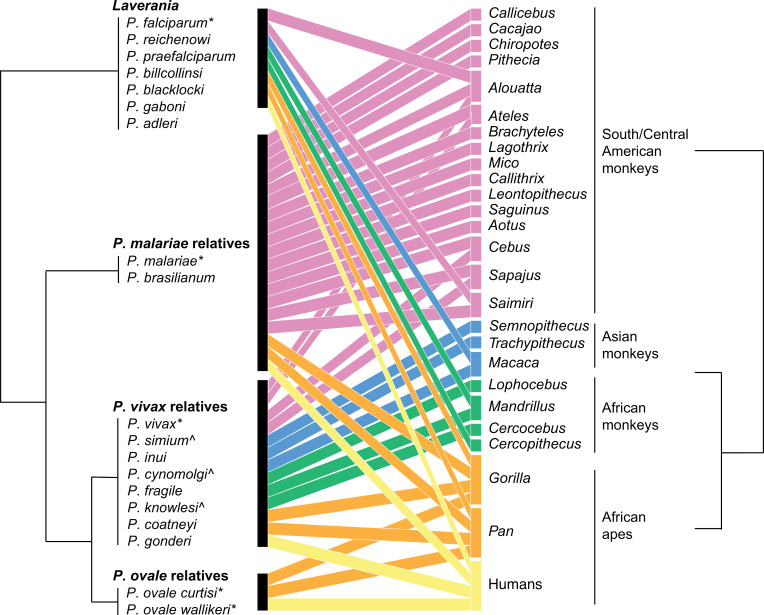
Bipartite plot of malaria parasite clade infection in primate genera. Phylogeny of malaria parasites follows Sharp et al., 2020, with parasites grouped by their clade based on the primary human-infecting parasite in that clade; clade names are above parasite groups. Branch lengths are arbitrary. Colors correspond to the primate clade. Figure made in R bipartite package (Dormann et al., 2008). * denotes common human-to-human-transmitting parasites, and ^ denotes nonhuman primate (NHP) parasites that have been found to naturally infect humans.

We focus on primate malarias because of NHPs’ close evolutionary relationship to humans and known parasite sharing with humans that produces disease. Therefore, primate-*Plasmodium* relationships provide an important system to demonstrate the links between ecological and evolutionary perspectives with direct medical relevance. To better predict future zoonoses or to build interventions for ongoing zoonoses, the drivers of NHP to human host switching have been a focus of empirical and mathematical modeling studies (Abdullahi et al., 2013; Imai et al., 2014; Yakob et al., 2018, Medeiros-Sousa et al., 2021). Starting with *P. knowlesi* in the 1930s, controlled experimental studies have confirmed a variety of NHP malaria parasites can infect human hosts, and epidemiological and genetic studies have confirmed a subset cause infection in natural settings (Knowles and Gupta, 1932, Eyles et al., 1960; Coatney et al., 1961; Schmidt et al., 1961; Chin et al., 1968, Contacos et al., 1963; Deane et al., 1966; Coatney et al., 1966; Contacos et al., 1970). Yet, we are only beginning to understand the extent of zoonotic malaria cases, the rate of human-to-human transmission, and the ecological and evolutionary factors that underlie the origin and spread of *Plasmodium* across primate hosts.

## 2. Database

To examine the ecological, evolutionary, and sampling processes that underlie the host specificity of primate malaria parasites, we collated an occurrence database of published records of the location and species involved in wild primate infections. We also collated a database of NHP *Plasmodium* species occurring in humans. Evidence for malaria in lemurs is limited, with no whole-genome sequences available, so we focus on Central/South American monkeys, Asian and African monkeys, and apes.

### The NHP malaria database

We build on the database published in Faust and Dobson, 2015 and the Global Mammal Parasite Database (Nunn and Altizer, 2005; Stephens et al., 2017). We combined the two databases and updated them with new publications from January 2015 to August 2020. Following the methods of Faust and Dobson, 2015, we searched the terms ‘*Plasmodium’* followed by each ‘genus of primates’ in PubMed and Web of Science between January 2015 and August 2020. For each publication, we recorded the name of the host species, the location of sampling, the number of individuals sampled, the sampling method (fecal or blood), the number of individuals infected, and the *Plasmodium* species found. We followed the phylogenetic naming used by Faust and Dobson, 2015 for the database and for search terms.

### The human zoonotic malaria database

We next built a database of NHP *Plasmodium* species sampled from humans; that is, zoonotic malaria occurrences. We followed a similar approach, searching PubMed and Web of Science for studies published between January 2015 to August 2020 with the search terms ‘name of each *Plasmodium* species naturally found in primate populations’ followed by ‘human.’ For each publication, we recorded the location of infection when indicated (or the location where the blood sample was taken if unavailable), the number of individuals infected, and the *Plasmodium* species. We focus on this time period because molecular methods have dramatically changed the taxonomy and identification of zoonotic malaria, and we aim to avoid misclassification from early studies. Additionally, given the recent rise in sampling, we expect that this time period captures the vast majority of zoonoses.

## 3. The origin of human-infecting malaria: Zoonotic malaria is a major human health burden across timescales

Here, we review the origin and current status of the *Plasmodium* parasites that regularly infect humans in natural settings, discussing the importance of transmission from NHPs to humans across timescales.

### Ancient zoonoses maintained today by human-to-human transmission

*P. falciparum* and *P. vivax* are responsible for approximately 95% of all malaria infections in human populations today (WHO, 2017). Substantial progress in the last decade has filled out the *Plasmodium* phylogeny and informed the timing and host origin of these two species. Yet, large questions about parasite origins remain; the direction of host switching based on modern sample diversity can be unclear given a lack of model-based inference, and sampling is still limited for wild, often endangered, NHPs. Both parasites have likely been circulating in human populations for thousands of years. *P. falciparum* is inferred to have switched into humans ~10,000–50,000 years ago (Prugnolle et al., 2011; Otto et al., 2018), and the higher diversity in *P. vivax* supports an older host switch (Neafsey et al., 2012). Despite their zoonotic origin, today, these parasites are maintained by human-to-human transmission, likely with little input from original reservoirs and substantial evolution since the host switch occurred (Pearson et al., 2016; Loy et al., 2017; Sharp et al., 2020).

Given the close phylogenetic relationship between *P. vivax* and multiple macaque malaria parasites, the primary hypothesis for many years was that *P. vivax* emerged in ancient human populations from macaques in Southeastern Asia (Escalante et al., 2005; Neafsey et al., 2012). Consistent with an out of Southeast Asia serial founder effect, recent analyses of genomewide variation in global isolates of human *P. vivax* show increasing linkage disequilibrium and decreasing diversity with distance from Asia (Daron et al., 2020). Recent findings of *P. vivax*-like parasite in wild African chimpanzee populations have questioned this long-standing hypothesis, proposing an African ape origin for *P. vivax* (Liu et al., 2010; Liu et al., 2014; Loy et al., 2017). Incomplete lineage sorting, and perhaps sampling biases, has made phylogenetic inference difficult, with support for contrasting placement of *P. vivax* as both a sister group to ape *P. vivax* and for it as a subset of ape parasite radiation (Daron et al., 2020; Sharp et al., 2020). A high frequency of the Duffy-negative allele in Africa, which is highly protective against *P. vivax* infection, may support an African origin. However, interpreting host adaptations as evidence of parasite origin is complicated because the occurrence and distribution of adaptive variants are limited by multifaceted pressures such as population size, available genetic variation, and random mutation. Models of the origin of Duffy-negative in sub-Saharan Africa suggest it rose in frequency only ~42,000 years ago (McManus et al., 2017), perhaps weakening the evidence for long-term coevolution in the region. Both Asian- and African-origin hypotheses remain plausible, and more geographic sampling, higher-quality genomes, and clearer analytical inference will be needed to differentiate between these alternative scenarios.

*P. falciparum* was historically thought to be inherited from a common ancestor of humans and chimpanzees, which then coevolved with their respective hosts into human *P. falciparum* and chimpanzee *Plasmodium reichenowi* (Escalante and Ayala, 1995; Loy et al., 2017). Extensive sampling of great ape parasites using noninvasive fecal sampling has demonstrated the deep and previously unappreciated diversity of ape parasites in the subgenus *Laverania*, the closest relatives to *P. falciparum*. The huge radiation of human *P. falciparum* is currently inferred to completely fall within the tree of the gorilla *Plasmodium praefalciparum*, interpreted as a recent African-ape-origin of the deadliest human malaria parasite, perhaps in the last ~10,000–50,000 years (Liu et al., 2010; Prugnolle et al., 2011; Otto et al., 2018; Sharp et al., 2020). A recent origin is also supported by the low levels of genetic diversity observed in global isolates of human *P. falciparum* compared to other *Laverania* species and to human *P. vivax* isolates. Other *Laverania* species have not been found in human populations, even in populations that overlap geographically with ape hosts (Sundararaman et al., 2013; Délicat-Loembet et al., 2015).

The other two parasites that historically and commonly cause malaria in humans, *P. malariae* and *Plasmodium ovale*, are less studied, cause fewer overall infections, and less severe disease (Rutledge et al., 2017). *P. malariae* has close relatives in both African apes (*Plasmodium rodhaini*) and South American monkeys (*P. brasilianum*) (Collins and Jeffery, 2007). The direction of host switching remains unclear, and only limited whole-genome sequence data is available (Sharp et al., 2020). However, the high diversity and presence of other divergent lineages related to *P. malariae* within African apes support an ancient African origin followed by a recent switch from humans to South American monkeys, perhaps during the Transatlantic slave trade (Rayner, 2015; Rutledge et al., 2017). Relatives of *P. malariae* seem to readily infect a variety of primate hosts (Figure 1), and may represent a single species.

The human parasite *P. ovale* consists of two subspecies *P. ovale curtisi* and *P. ovale wallikeri,* and is mostly found in Malaysia and Africa (Duval et al., 2010; Rutledge et al., 2017). African apes harbor nearly identical *P. ovale curtisi-*like and *P. ovale wallikeri-*like populations*,* suggesting that the two human *P. ovale* species may have diverged in apes before their spread into human populations. However, these *P. ovale* parasites seem to occur very infrequently in apes in the wild, and limited genomic data is available making the direction and timing of host transfer unclear.

### Ongoing zoonoses, with unknown human-to-human capability

In addition to the ancient zoonoses that founded modern human-infecting malaria species, multiple NHP malaria parasites are currently infecting humans, particularly in Southeast Asia and South America. Evidence of ape-to-human transmission of *Laverania* in Africa is rare, but has been documented (Ngoubangoye et al., 2016).

**Figure 2.**
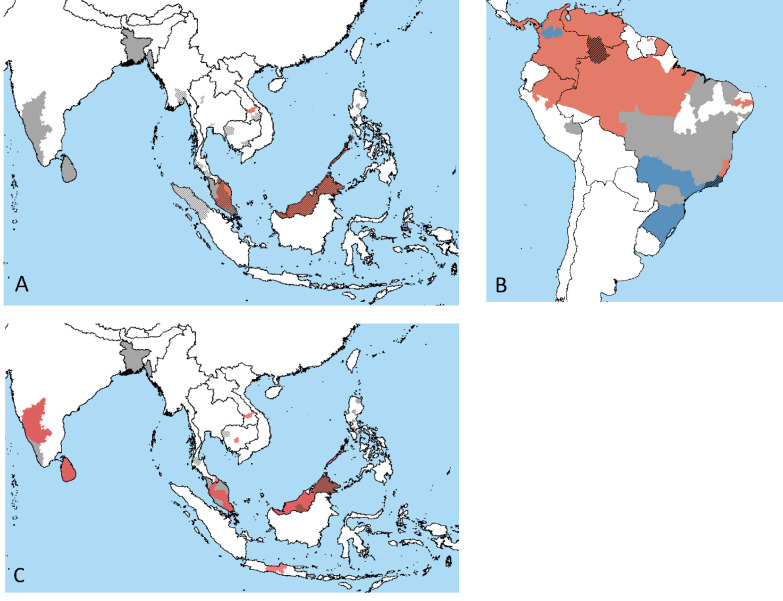
Geographic distribution of emerging zoonotic malaria cases. The solid colors represent cases occurring in nonhuman primates (NHPs), the gray solid color represents the areas where NHPs have been tested for *Plasmodium* species but none were identified, and black hatching represents cases in human, often overlapping NHP ranges. Presence of (**A**) *P. knowlesi* (orange) in Southeast Asia, (**B**) *P. brasilianum* (orange), and *P. simium* and *P. brasilianum* (blue) in South America and (**C**) *P. cynomolgi* (orange) in South and Southeast Asia. Data available in supplement, with data collation methods described in Section 2. Image made in Google Earth.

Today, zoonotic *P. knowlesi* is the most frequent malaria-causing agent in Malaysia and is widespread in Southeast Asia from its macaque origin (Figure 2B; Rajahram et al., 2019; WHO, 2014, WHO, 2019). Despite its high incidence, evidence for human cycles of transmission has not been clearly demonstrated, but is presumed. Genetic data suggests divergent populations of *P. knowlesi* circulating amongst different macaque species (Divis et al., 2015). Previously misdiagnosed as both *P. vivax* and *P. malariae*, the timing of *P. knowlesi* first shifting into humans is unclear (Singh et al., 2004). Though less common and well-understood, occasional natural infections of another macaque parasite, *Plasmodium cynomolgi*, have also been observed in Southeast Asia (Ta et al., 2014). The high rates of *P. cynomolgi* in the wild suggest undiagnosed current or future risk of zoonosis is possible (Zhang et al., 2016).

**Figure 2—figure supplement 1.**
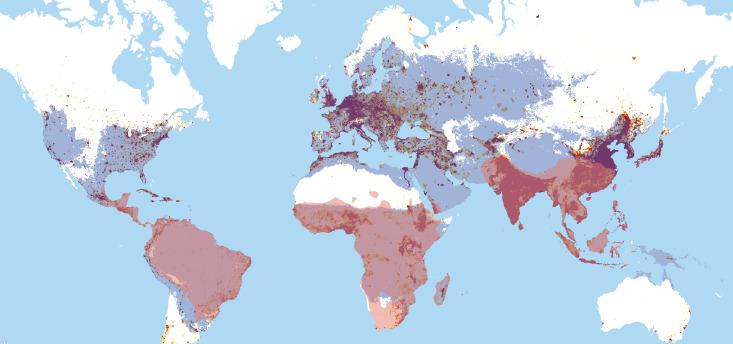
Geographic distributions of hosts and vectors for zoonotic malaria. The regions colored in blue correspond to the presence of the vector *Anopheles*. The regions colored in pink correspond to the presence of nonhuman primate (NHP) hosts. The regions overlapping the presence of mosquitoes, NHPs, and humans are potentially areas of increased concern for cross-species malaria transmission. The human density is from the Center For International Earth Science Information Network-CIESIN-Columbia University, 2018; NHP ranges are from IUCN, 2020. *Anopheles* range is from Sinka et al., 2012. Image made in Google Earth Engine (Gorelick et al., 2017).

South America is a newly identified hotspot for emerging zoonotic malarias, though the timeline and extent of zoonoses remain unknown because of a lack of historical or archival sampling. Early experimental studies confirmed the possibility of human infection of NHP malaria parasites *P. brasilianum* and *P. simium* (Contacos et al., 1963; Deane et al., 1966). The first natural infections in humans were only described in 2015 and 2017 for *P. brasilianum* and *P. simium*, respectively (Lalremruata et al., 2015; Brasil et al., 2017). Because of their close morphological and genetic relationships to common human-infecting malaria parasites, previous zoonotic infections of *P. brasilianum* and *P. simium* have been misclassified as *P. malariae* and *P. vivax*, respectively. Indeed because of their great genetic, morphological, and immunological similarity, it is unclear that the pairs *P. brasilianum* and *P. malariae*, or *P. simium* and *P. vivax*, should be classified as different species. Therefore, these cases may be either true zoonoses or examples of the host generalism of *P. malariae* relatives and *P. vivax* relatives.

Genomic data provides some support for differentiation between *P. simium* and *P. vivax* (Mourier et al., 2021; de Oliveira et al., 2021), but is weaker for *P. brasilianum* and *P. malariae* comparisons, which lack whole-genome data. Currently, most South American *P. malariae* cases are linked to international travel, but NHPs may serve as a reservoir for disease (Figure 2). Similarly, extra-Amazonian cases of *P. vivax* have been rising in Brazil (Brasil et al., 2017), and further testing will be required to determine if some of these are misdiagnosed zoonotic *P. simium* infections. Further genetic and epidemiological studies are needed to clarify the extent of natural human infection, the taxonomic relationships, and determine if their immediate origin is transmission from NHPs or from other humans. For example, widespread genotyping of malaria cases presumed to be *P. vivax* in humans can establish the geographic range and frequency of *P. simium* infection, and a combination of mathematical modeling and contact tracing or other public health surveys would inform the likelihood of human-to-human transmission versus recurrent monkey-to-human transmission. Additionally, recent identification of *P. simium* in other regions shows the importance of further geographic sampling to understand the origin of *P. simium*, including the timeline, location, and number of introductions (Rondón et al., 2019).

## 4. Increasing threat of human malarias infecting NHPs with subsequent risk for humans

Malaria parasite sharing is not unidirectional; increasing human pressures on local NHP populations are reintroducing human *Plasmodium* species into other primates, putting often endangered species at further health and conservation risk. Additionally, these hosts may become reservoirs for human malaria, with these parasites later transmitting into human populations or other NHPs.

Because the great apes have close relatives of human-infecting *Plasmodium* circulating in their populations, it is hard to identify anthroponotic infections (i.e., transmission of human endemic parasites to other species). However, the presence of drug-resistant mutations can be used to predict the direction of transfer as human to ape. Using this evidence, the human *P. falciparum* has been found recurrently in primate populations living near humans (Ngoubangoye et al., 2016; Prugnolle et al., 2013; Loy et al., 2017). *P. vivax*, *P. malariae*, and *P. ovale* have also been occasionally, but rarely, described in African apes (Kaiser et al., 2010; Duval et al., 2010; Hayakawa et al., 2009; Rayner et al., 2011; Sharp et al., 2020). Further whole-genome sequencing or typing of known diagnostic regions will be able to differentiate between these close parasite relatives and inform the risk level of anthroponoses.

South American malaria parasites demonstrate the risk to humans of anthroponoses from wild NHPs. Now considered a zoonotic malaria parasite, *P. simium* is proposed to have originated as a human-to-howler monkey switch of *P. vivax* during European colonization and the Transatlantic slave trade in Brazil. An early hypothesis suggested that *P. vivax* come from a pre-Columbian introduction 15,000–30,000 years ago as humans first arrived. But new methodologies and a better sampling have supported a recent introduction, ~500 years ago with European colonization (Culleton et al., 2011; van Dorp et al., 2020; Taylor et al., 2013; Hupalo et al., 2016; Rodrigues et al., 2018). Historical DNA from pre-eradication Spain suggest a recent introduction and close relationship between historical Southern European *P. vivax* and modern South American *P. vivax* (van Dorp et al., 2020). This is consistent with a host switch of historical *P. vivax* into howler monkeys to become what is today known as *P. simium*, supported by the low genetic diversity of *P. simium* and high similarity morphologically and genetically to *P. vivax* (Escalante and Ayala, 1995; Lim et al., 2005; Mourier et al., 2021; de Oliveira et al., 2021). Since then, *P. simium* has built up a handful of genetic differences from *P. vivax*, perhaps through drift or adaptations to a new host (Brasil et al., 2017; Mourier et al., 2021; de Oliveira et al., 2021). Occasional cases of *P. falciparum* have also been reported in South American monkeys, though these cases are rare and largely unconfirmed (Duarte et al., 2008; Yamasaki et al., 2011).

The history of *P. malariae*/*P. brasilianum* in the Americas is similarly complicated by sharing across multiple primate hosts. Today, the classification of these two parasite species seems to follow the host in which they are found *– P. malariae* for humans and *P. brasilianum* for NHPs – rather than parasite characteristics (Lalremruata et al., 2015). *P. malariae/brasilianum* is incredibly widespread in South American monkeys and appears to circulate freely between primate and human populations and be a single anthropozoonotic species in South America. Similar to *P. simium*, the presence of *P. malariae/P. brasilianum* in American NHPs likely originated with a human-to-primate transmission associated with the Transatlantic slave trade, from African *P. malariae* (Collins and Jeffery, 2007; Rutledge et al., 2017; Lalremruata et al., 2015). *P. malariae* is now rare in humans in South America, with most cases either introduced by international travel or potentially through new zoonoses from primate reservoirs.

## 5. Investigating distributions of parasite sharing among hosts

A variety of methods have been used to investigate the distribution of parasites among hosts. One starting point is to produce a matrix of hosts and the parasites (Cooper et al., 2012), which is known as an incidence matrix in ecology. Another approach maps the occurrence of a parasite onto a phylogeny (Cooper et al., 2012), or compares host and parasite phylogenies, aiming to identify and visualize host shifts along with parasite duplications (i.e., within host speciation) and parasite extinctions (Charleston, 1998; Huelsenbeck et al., 2000, Ricklefs and Fallon, 2002; Garamszegi, 2009). Phylogeny is often a strong predictor of parasite sharing because of shared physiological, genetic, and environmental factors. Finally, a number of authors represent host-parasite incidence data as a bipartite network (Poulin, 2010). As its name suggests, a bipartite network has two parts: one for hosts and another for parasites (Figure 1). Edges are placed between organisms in each part (but not within) based on the occurrence of a parasite in a host. One can then generate a unipartite projection of this bipartite network showing how hosts are connected through the parasites they share (or parasites are connected through the hosts that they share) (Gómez et al., 2013). A major risk in all of these approaches is that we rarely know all of the parasites in a collection of hosts, with some parasites or hosts studied better than others (Walther et al., 1995; Stephens et al., 2016). For example, terrestrial primate species are more likely to be sampled for parasites than arboreal primate species (Cooper and Nunn, 2013; Poulin et al., 2016). Thus, a variety of approaches have been developed to deal with variation in sampling effort (Nunn et al., 2003; Elmasri et al., 2020; Teitelbaum et al., 2020; Amoroso and Nunn, 2021).

The range of hosts that a parasite infects can also be quantified using measures of phylogenetic host specificity (Poulin, 2010; Cooper et al., 2012). This concept is important in the context of human and animal health because it determines the potential for cross-species transmission, with phylogenetic host specialists generally only crossing narrow phylogenetic distances, while phylogenetic host generalists can cross a wider phylogenetic range of hosts. Thus, for emerging zoonoses in humans, we should be concerned about phylogenetic host specialists arising from great apes and other NHPs and phylogenetic host generalists in other mammals. In one recent study, Park et al., 2018 used a database of >1400 parasite species and 404 mammal host species to quantify phylogenetic host specificity and its correlates. They found that arthropods and bacteria are the most generalist, viruses and helminths are intermediate in generalism, and protozoa are the most specialist of the parasites in this database. Park et al., 2018 also found that close-contact transmission is most associated with specialization on fewer hosts. These analyses also revealed a pattern consistent with a ‘leaps and creeps’ strategy by parasites, with some parasites mostly infecting closely related hosts, but occasionally taking a ‘leap’ to less related hosts, where the parasite circulates again amongst close relatives. For primate malarias, cross-species transmission, and therefore parasite sharing among hosts, is a confluence of factors in the parasite, vector, and multi-host system, and is a product of both their geography and phylogeny.

### The geographic distribution of natural and experimental zoonotic malaria

Figure 2 plots the primate and human presence of zoonotic malarias *P. simium*, *P. knowlesi*, and *P. cynomolgi*. Publications where samples were taken from NHPs in zoos, breeding farms, or otherwise with no known origins were excluded from the figures.

Figure 2A and B show the distribution of *P. knowlesi* and *P. cynomolgi* cases, respectively, in both humans and NHPs. Reported cases of *P. knowlesi* in humans have occurred throughout Southeast Asia, including Malaysia, Lao, Myanmar, Indonesia, Cambodia, and Thailand, whereas primate infections have primarily been documented in Malaysia. The risk of zoonosis throughout Southeast Asia is variable owing to both a lack of sampling (Figure 2) and potential misdiagnosis as common human-infecting malarias (Shearer et al., 2016; Zhang et al., 2016). Given primate and vector ranges throughout the region, the lack of *P. knowlesi* is more likely due to undersampling of primate parasites than their absence. *P. cynomolgi* is present in primates throughout South Asia, though the range often does not overlap with known human infections (Figure 2C; Zhang et al., 2016; Imwong et al., 2019; Grignard et al., 2019; Hartmeyer et al., 2019; Raja et al., 2020). Similar to *P. knowlesi*, this suggests that variation in sampling effort drives the estimated species distributions, especially outside Malaysia, which has invested heavily in sampling efforts because of its high *P. knowlesi* burden.

Other *Plasmodium* species naturally found in primate populations in Southeast Asia have been experimentally transmitted to humans, such as *Plasmodium inui* (Coatney et al., 1966), but have not been observed naturally. Such cases are worth monitoring for potential parasite genetic mutations or environmental changes that facilitate new zoonoses. *P. inui* is a strong candidate for future zoonotic transmission because it shares a host, some potential vectors, and environment with the known zoonotic parasites *P. cynomolgi* and *P. knowlesi* (Baird, 2009; Coatney et al., 1966; Maeno et al., 2015).

In South America, *P. malariae* and *P. brasilianum* are found throughout the continent and are able to infect a large number of primate species, whereas *P. simium* is known primarily from the Atlantic Forest region in Southeastern Brazil (though there have been recent reports in Colombia as well), infecting only a few American NHPs (Figures 1 and 2b; de Alvarenga et al., 2015, Brasil et al., 2017; Rondón et al., 2019). Currently, known outbreaks of primate *Plasmodium* in human populations are from a limited number of locations; however, the presence of *P. malariae/P. brasilianum* throughout much of South America suggests a wider distribution of human infections is possible, and may already be occurring unreported since these parasites produce less severe disease (Lalremruata et al., 2015; Brasil et al., 2017).

More generally, the geographic distribution of zoonotic malaria is influenced by the distributions of NHP, human, and mosquito ranges. Figure 2—figure supplement 1 plots the ranges of both hosts and vector to inform areas that may be at increased risk of future zoonoses – those with overlap in the range of NHPs, humans, and mosquito vector. Comparing Figure 2 and Figure 2—figure supplement 1, we see multiple regions with zoonotic malaria transmission despite low human population densities. That is, zoonotic malaria emergence may be more closely linked to the overlapping presence of NHP and vector populations. Additionally, many regions of overlap have not been thoroughly sampled; therefore, the amount of cross-species transmission may be underestimated.

### Phylogenetic distributions of *Plasmodium* sharing among primates

Using the databases of the occurrence of *Plasmodium* species in primates presented here, Figure 1 shows a bipartite plot to visualize malaria parasite sharing among primate hosts with host and parasite phylogenies at the genus and subgenus level, respectively. We include scenarios of natural infection observed in the wild.

At the species level, *Laverania* species are primarily primate genus-specific, with *P. reichenowi*, *Plasmodium billcollinsi*, *Plasmodium billbrayi*, and *Plasmodium gaboni* present in chimpanzees, and *P. praefalciparum*, *Plasmodium blacklocki*, and *Plasmodium adleri* present in gorillas. In contrast, *P. malariae-*related and *P. vivax-*related species often infect many NHP where they occur. At the subgenus level, most parasites appear to be generalists, infecting diverse primate families, except for *P. ovale* relatives who are found only in great apes. The difference in specialization observed at different taxonomic levels is partially driven by recent human to NHP parasite sharing of parasites, notably of *P. falciparum* in South America.

Consistent with the pattern of specialist *Laverania* and generalist *P. vivax* relatives, human *P. falciparum* infects fewer NHPs than *P. vivax* relatives, and few *Laverania* species have been observed in humans today (but see Ngoubangoye et al., 2016). The reason for *Laverania* specialization is unclear, however, because *Laverania* ancestral species switched to become the most widespread human-infecting malaria historically, *P. falciparum*. The consequence of host specialism for human infection rates is not necessarily directly related to the frequency at which parasite species switch hosts. Even potentially rare host switching of *Laverania* has led to dramatic consequences for humans, as seen with the evolutionary success of *P. falciparum*. In contrast, multiple *P. vivax* relatives have been found to naturally infect humans, including *P. knowlesi*, *P. cynomolgi*, and *P. simium*, yet only *P. vivax* itself is widespread (with potentially increasing infection of *P. knowlesi*).

Overall, the few numbers of subgenera make it difficult to draw strong conclusions about the relationship between phylogeny and host specificity, particularly at different taxonomic scales. For example, while *P. vivax* relatives are generalists infecting all primate families considered here, substantial variation within lower taxonomic levels is observed; *P. simium* and *P. vivax* have only been found in a handful of South American monkey genera despite extensive sampling (Figure 2), and *P. vivax* is rarely observed in African monkeys, though its relative within the same subgenus relative *Plasmodium gonderi* infects them. Notably, parasites from across the *Plasmodium* phylogeny regularly infect human populations. In particular, the two most common human-infecting malaria parasites, *P. falciparum* and *P. vivax*, are from highly divergent parts of the *Plasmodium* phylogeny and are thought to represent independent zoonoses.

Though host specificity appears correlated with phylogenetic relationships, the difference in host specificity may derive from ecological causes instead of or in addition to phylogenetics. The phylogeny of *Plasmodium* largely corresponds to geographic regions where the parasites are found – with *P. vivax*-related more common in Asia and South America, and *P. falciparum* and *Laverania* spp. in Africa – making the effects of phylogeny and geography difficult to disentangle. This geographic division between parasites may also have differed historically given recent findings of *P. vivax*-like parasites in African apes (Prugnolle et al., 2013; Liu et al., 2014). Further sampling and population genomic studies should aim to clarify the timing of these relationships. Faust and Dobson, 2015 identified species and regions that are likely undersampled with respect to parasite diversity, suggesting that new primate malaria parasites may occur in African monkeys and lemurs. Updating their database here, we see that even areas that are predicted to be unlikely to introduce new parasite species, such as Southeast Asia and South America, still have substantial undersampling of known parasite diversity and range (Figure 2).

### Quantifying host-parasite co-occurrence

Previous work has quantitatively interpreted visualizations of parasite sharing among hosts, attempting to control for geography. For example, Garamszegi, 2009 tested whether malaria parasites of the same taxonomic group tend to infect primate hosts of similar taxonomic group or geographic region. Co-occurrence between host and parasite genera suggests a history of co-divergence and host specialization, whereas large differences may suggest recent host switching or host generalism. They found an association between host and parasite phylogenies consistent with co-divergence at the family level, though the result was not statistically significant at the genus level; parasites tended to infect closely related hosts. Following the methods of Garamszegi, 2009 equations 1 and 2 (originally from Ricklefs and Fallon, 2002) and using our database of parasite occurrence in primate hosts presented here, for the eight human-infecting *Plasmodium* species, we calculate a probability of drawing two hosts from the same genus and continent as H=0.06, with p=0.07 (significance from binomial probability). This is qualitatively similar to the result of Garamszegi, 2009, who found H=0.11, with p=0.19 considering both human and NHP malarias and a slightly different taxonomy.

Quantitative analyses such as these will be important to understand the drivers of parasite distributions among hosts moving forward. However, major barriers exist to application and interpretation of these methods directly to the currently available data. These barriers include uncertainty in parasite phylogenetic relationships, incomplete host sampling in the wild, and perhaps most critically, inconsistencies in parasite species naming that are often confounded or defined by the host in which they are sampled. Additionally, primate phylogeny is strongly related to geography and comparisons within a geographic region substantially reduce sample size.

## 6. Factors driving host sharing and specificity

The observed patterns of *Plasmodium* parasite sharing among primate hosts – described above – are shaped by a variety of ecological and evolutionary factors impacting the parasite, host, and vector. Understanding the primary mechanisms behind these parasite distributions will be important for predicting parasite emergence in human populations and designing effective interventions.

For many parasites, phylogeny is a major predictor of host sharing: more closely related species are expected to harbor the same parasite because they share underlying physiology, immune defenses, behavior, and in the case of intracellular parasites such as viruses or some protozoa, these hosts share similar cellular phenotypes, such as viral or parasite entry receptors (Woolhouse, 2002; Longdon et al., 2014). This is expected to generate phylogenetic signal in host and parasite infection patterns and to influence probabilities for shifts among different hosts. The effect of phylogeny has been documented in primates, with research revealing that more closely related primate hosts have more similar parasite communities (Davies and Pedersen, 2008, Cooper et al., 2012). Similarly, a study of rabies virus among North American bat species also found support for higher probability of host shifts among more closely related species of bats (Streicker et al., 2010), while an experimental study demonstrated an effect of phylogeny on viral titers for three viruses in *Drosophila* (Longdon et al., 2011). This phylogenetic relationship has been observed in avian and primate malarias (Ricklefs and Fallon, 2002; Garamszegi, 2009), and we consider support for it in Section 5.

In addition to phylogenetic relatedness, geographic overlap and host phenotypic and ecological characteristics also predict parasite sharing (Clark and Clegg, 2017). Hosts that share parasites must overlap geographically with one another (or with some other host that shares a vector and acts as a reservoir to infect them both); both geographic and phylogenetic effects have been observed in primates (Davies and Pedersen, 2008; Cooper et al., 2012). Across mammals, Albery et al., 2020 found that both phylogeny and geographic overlap predicted sharing of viruses. Traits that lead to direct or indirect interactions between species, such as a shared diet, water source, or sleep site, will also facilitate parasite sharing. A variety of evidence supports these effects. In a study of primates, for example, Cooper et al., 2012 found that similarity in body mass predicted the similarity of parasite communities in pairs of species (together with effects of phylogeny and geography). In a study of bat viruses, Willoughby et al., 2017 found that cave-roosting increases parasite sharing, along with geographic overlap and phylogenetic distance. Anthropogenic change is influencing many of these processes, with likely consequences for human disease risk and primate conservation. Also important, but less well studied in natural systems, are the daily rhythms of hosts, vectors, and the different life stages of parasites (Owolabi et al., 2021; Prior et al., 2020). Here, we highlight key ecological and evolutionary factors such as these that drive the distribution of parasites across hosts for the case of primate malarias.

### Ecology, environment, and behavior shape the distribution of malaria among primates

An important ecological predictor of cross-species *Plasmodium* transmission is having shared vectors. This requires that the vector exhibits preferences for both host species and that the hosts overlap geographically. For example, the parasite with the highest disease burden in humans is spread by a group of *Anopheles* vectors, *Leucosphyrus*, known to bite both humans and other primates (though some species do show host preferences) (Galinski and Barnwell, 2009). *Anopheles cracens*, found mostly in peninsular Malaysia, is known to bite humans and NHPs, with some preference for humans. It is considered as one of the main vectors of *P. knowlesi* in human populations (Vythilingam et al., 2008; Jiram et al., 2012; Lau et al., 2016). The vectors of *P. knowlesi* include *Anopheles balabacensis* and *Anopheles latens*, found in Sabah and Sarawak, respectively. All of these vectors are commonly found in the forest or surrounding peri-domestic areas, implicating certain times and locations where transmission to humans is more likely to occur (Tan et al., 2008; Manin et al., 2016).

Multiple aspects of climate, including temperature, precipitation, and humidity, influence the life cycle of mosquito vectors and their geographic range, and thus can influence parasite sharing. For each developmental stage of the mosquitoes – from eggs to adults – the temperature is one of the most important factors that influence the duration of the passage from one stage to another; climatic change is likely to alter the distribution of the vector across latitudes and altitudes, and therefore the disease geography (Shapiro et al., 2017; Depinay et al., 2004; Mordecai et al., 2013; Mordecai et al., 2019). Because the parasite develops inside the mosquito’s salivary glands, the temperature also plays a role in parasite viability (Shapiro et al., 2017). Although the parasites and vectors respond strongly to temperature changes, the outcomes can be negative or positive (Christiansen-Jucht et al., 2014). Climate change is often predicted as enhancing the transmission of mosquito-borne disease, although the consequences are often nonlinear (Reiter, 2001; Mordecai et al., 2013; Franklinos et al., 2019). The global ecology of mosquito and host can be used to better predict future geographic distributions of disease risk (Mordecai et al., 2013; Mordecai et al., 2019; Shapiro et al., 2017; Depinay et al., 2004; Ryan et al., 2019; Carlson et al., 2021).

Anthropogenic change, including deforestation, urbanization, demographic expansion, and hunting, is also believed to increase frequency of interaction with animal populations and therefore to induce a higher risk of zoonosis (Murray and Daszak, 2013; Wolfe et al., 2005; Loy et al., 2017; Plowright et al., 2017; Mwangi et al., 2016). These processes have been linked to increased malaria transmission; however, urbanization may also decrease malaria incidence by removing mosquito habitats and increasing health infrastructure (De Silva and Marshall, 2012; Wang et al., 2020; LaDeau et al., 2015). For example, increasing demand for palm oil has reshaped land use patterns throughout Southeast Asia, increasing deforestation and potentially changing the distribution of vectors and hosts for *P. knowlesi* (Vijay et al., 2016). In Malaysia, deforestation and habitat loss have pushed macaque populations closer to farms and semi-urban areas, where the vector *Leucosphyrus* occurs, leading to close contact between humans, NHPs, and vectors (Vythilingam et al., 2018, Sueur et al., 2019). Fornace et al., 2019 found that land use change such as disturbed forests and agriculture are the main factors associated with zoonotic transmission of *P. knowlesi*. Indeed, the main *P. knowlesi* vectors and NHP host (*Macaca*) occur in disturbed forest where human populations are more likely to be found (Moyes et al., 2016, Fornace et al., 2019). Forest loss can also lead to other environmental changes, such as an increase in local temperature or a modification of breeding sites, increasing malaria transmission (MacDonald and Mordecai, 2019; Yasuoka and Levins, 2007). The relationship between deforestation and malaria incidence is complicated and multidirectional because increases in human occupation of forested areas will initially increase exposure, though with time larger settlements often provide improved access to healthcare and urbanization. MacDonald and Mordecai, 2019 found that deforestation increases malaria prevalence but that increasing malaria prevalence is associated with reduced forest clearing and economic activity in Brazil; this bidirectional relationship highlights the need for location-specific modeling to understand the drivers of parasite distributions and infection rates.

Asymptomatic cases, widely documented for human malaria species especially for *P. vivax* and *P. falciparum*, can contribute to the persistence of epidemics and act as a reservoir in human populations (van Eijk et al., 2019; Cheng et al., 2015; Okell et al., 2009), and likely for *P. knowlesi* (Fornace et al., 2016). The role of asymptomatic cases in further human-to-human transmission of zoonotic malarias is unclear and will be important to understand transmission dynamics and ideal control strategies.

Ecological competition is also increasingly being appreciated as a driver of malaria parasite case distributions. As we make needed progress on *P. falciparum* elimination, the potential for zoonotic malaria to fill open niches must be monitored. This trade-off may have occurred with elevated rates of *P. vivax* in regions with declining *P. falciparum*. A similar trade-off has also been proposed for the rise of *P. knowlesi* (William et al., 2014; Cooper et al., 2020) as rates of *P. vivax* and *P. falciparum* decline. Monitoring open ecological niches will be important for preventing new emergences or resurgence of other malaria parasites.

### Host-parasite genetic interactions shape the distribution of malaria among primates

Potentially underlying the co-divergence patterns observed in phylogenies is the coevolution of host-parasite interactions, which determines which parasites may be able to infect which hosts. Between-species variation in malaria susceptibility is seen in NHPs, supporting a role for genetics in susceptibility. For example, rhesus macaques exhibit more severe disease from *P. knowlesi* than long-tailed macaques. These differences in morbidity may influence the geographic distribution of the hosts and possibly impact the macaque-to-human transmission (Coatney et al., 1961; Wheatley, 1980), though the genes involved are unknown. Here, we review genes and pathways in primates and in *Plasmodium* that are hypothesized to play a role in host-parasite interactions, focusing on those most likely to impact host specificity. The genetic basis of host-parasite interactions for human malarias has revealed multiple pathways that mediate host susceptibility and suggest coevolution. Central to these is protein interactions during invasion of host red blood cells, as well as immune evasion. This raises the question, are the same genetic pathways important for between-species malaria transmission?

Host red blood cells have a variety of surface proteins that mediate interactions with parasites during parasite invasion of host red blood cells (Lim et al., 2017; Su et al., 2020; Ebel et al., 2017; Leffler et al., 2017; Kariuki and Williams, 2020; Ebel et al., 2020; Band et al., 2021). For example, the Duffy antigen/chemokine receptor, *DARC* (also known as *ACKR1*), is a gene that encodes a surface glycoprotein. It is a classic example of adaptation in human evolution, with a single mutation producing the Duffy-null version of a surface glycoprotein that does not bind to *P. vivax*, preventing parasite invasion of host cells and most disease. Studies from multiple African populations have repeatedly found strong signatures of selection in human genomes at the *DARC* locus, likely because of its function in preventing *P. vivax* malaria (McManus et al., 2017; Kwiatkowski, 2005; Hamid et al., 2021; Pierron et al., 2018). This gene and its expression levels have also been implicated in parasite invasion or disease in multiple other primates with *P. vivax* relatives, including South American and African monkeys (Camargos Costa et al., 2015; Tung et al., 2009; McHenry et al., 2010; Gunalan et al., 2019; Trujillo and Bergey, 2020).

Another classic example of human adaptation involves resistance to *P. falciparum* malaria also through changes to red blood cell morphology, such as hemoglobin S (HbS), decreasing parasite invasion, and, in some cases, causing sickle cell disease and other hemoglobin disorders (Kwiatkowski, 2005; Ha et al., 2019; De Sanctis et al., 2017; Luzzatto, 2012; Kuesap et al., 2015). Additionally, transferrin receptor proteins are well-studied for their role in iron uptake by cells, but their role in parasite interactions is also increasingly being appreciated (Chan et al., 2020; Galinski et al., 1992; Gruszczyk et al., 2018). More generally, Ebel et al., 2017 found widespread phylogenetic signatures of adaptation in hundreds of genes that interact with malaria parasites.

Together, these results suggest that gene families important for host-parasite interaction may be shared across primates, with important insights gained from differences in species-specific variation, including a role for regulatory variation. With increasing sequencing of NHP genomes, an important next step will be to understand within- or between-species variation in these pathways across primates that may underlie host specificity and switching.

Parasites have complementary proteins that bind receptors on the surface of host red blood cells in order to enter host cells. These notably include Duffy-binding proteins (DBPs) and reticulocyte-binding proteins (RBPs) (Adams and Mueller, 2017; Lim et al., 2017; Su et al., 2020; VanBuskirk et al., 2004; Carlton et al., 2008). In vitro studies of *P. knowlesi* show that a junction does not form between host cell proteins and parasite when DBP is deleted (Singh et al., 2005). The DBP system in *P. vivax* shows incredible genetic diversity, including duplication events. However, *P. vivax* has recently been found to infect red blood cells of Duffy-negative people in Ethiopia and Madagascar, suggesting that alternative pathways to invasion exist (Ménard et al., 2010). Similarly, within-species genetic diversity across 11 genes in the *P. vivax* RBP family is hypothesized to mediate parasite-binding affinity (Lim et al., 2016), perhaps suggesting a role for between-species variation in host interactions.

Despite the importance of DBPs and RBPs in parasite invasion of host red blood cells, less is known about the role of these pathways in shaping host specificity and risk of host switching. Early genomic resources that derive from passage through monkeys are missing common RBP genes founds in human field isolates (Hester et al., 2013), suggesting potential for host specificity. The first whole genomes from *P. simium* support a role for DBPs and RBPs in parasite evolution to new hosts or host specificity (Mourier et al., 2021; de Oliveira et al., 2021). Without experimental studies to confirm function, caution is warranted. For example, population-genetic studies identified variants in RBPs that differed between human infections and those found in great apes; however, no significant difference in binding affinity was identified for parasites to great ape versus human red blood cells, suggesting that these RBP differences are not functionally critical for host specificity (Loy et al., 2018).

Multiple genes in red blood cell invasion pathways have also been implicated in the host specificity of *Laverania* parasites, and host switching of *P. falciparum* based on comparative genomic approaches (Martin et al., 2005; Rich et al., 2009; Rayner et al., 2011; Otto et al., 2018; Galaway et al., 2019; Plenderleith et al., 2019; Proto et al., 2019). Galaway et al., 2019 used ancestral sequence reconstruction for reticulocyte-binding-like homologous protein 5 (RH5) and quantified differences in binding affinity for human, gorilla, and chimpanzee cells. They found that the inferred ancestral protein had similar binding affinity for gorillas and humans, perhaps aiding in early transfer of the population that became modern *P. falciparum* in humans. This is in contrast with the modern *P. falciparum* version of RH5, which is highly human-specific (Wanaguru et al., 2013). Changes in the protein-binding systems for red blood cell invasion have also been implicated in *P. knowlesi* host switching (Moon et al., 2016; Dankwa et al., 2016).

In addition to red blood cell invasion, immune response is central to host-parasite interactions and coevolution. These incredibly diverse genes, such as AMA1, MSP1, CSP, and the *var* and *pir* gene family variants, bind to host receptors, are involved in immune evasion and have been associated with disease severity (Tetteh et al., 2009; Amambua-Ngwa et al., 2019; Conway, 2015; Neafsey et al., 2021). *var* genes have been identified in ape *Laverania* species, but their variation and impact on disease progression outside of humans is unclear, particularly because the corresponding ape immune response and genetic variation at immune genes is difficult to study in natural populations. Understanding the strain-specific immune responses and range of natural genetic variation is particularly important because *var* genes such as PfEMP1 have been proposed as vaccine targets, and expression of *var* genes has been shown to change in response to treatments (Bachmann et al., 2019; Jensen et al., 2020). Similarly, vaccines developed against *P. falciparum* have had mixed success based on the genetic diversity of parasites (Thera et al., 2011; Neafsey et al., 2015; Ouattara et al., 2015; Graves et al., 2016; Laurens et al., 2017; Bailey et al., 2020; Bell et al., 2021). Su et al., 2020 further discuss the role of these gene families in host-parasite interaction, and future work expanding to nonhuman malarias will be important to understand the role of these genes in between-species susceptibility and host switching.

Further sampling to understand host and parasite ranges and natural genetic variation, population-genetic studies of adaptation, and experimental work on the mechanisms of host specificity will inform whether natural parasite variation is able to bind with common host genes necessary for invasion and, therefore, predict primate malarias with the highest potential for establishment of disease in other species (Barber et al., 2017; Brasil et al., 2017; Assefa et al., 2015; Sharp et al., 2020; Faust and Dobson, 2015). Host and parasite variants in the associate proteins that interact on red blood cells determine the immunity induced; therefore, an understanding of natural variation across primates will inform future vaccine design (Chen et al., 2016; Su et al., 2020). Indeed, the high levels of genetic variation in *P. vivax* have been proposed as a barrier to an effective vaccine (Lim et al., 2017), and DBPs and RH5 have been proposed as vaccine targets for *P. vivax* and *P. falciparum*, respectively (Bustamante et al., 2013; Douglas et al., 2011; Camargos Costa et al., 2015; Chitnis and Sharma, 2008; Mueller et al., 2015; Chen et al., 2016; Su et al., 2020).

## 7. Moving forward: Emerging areas for research in primate malaria

Parasite transmission studies are often human-centric, focusing on animal-to-human transmission. Here, we emphasize a broader perspective that between-animal transmission also has direct medical and conservation impacts, and monitoring of NHP diseases should be a priority. This is particularly timely given recent conversations about long-term creation of reservoirs of SARS-CoV-2 in new animal populations, which could then serve as a new source for future evolution and spillover into humans.

A broader perspective on the role of humans in disturbing environments, changing climates, and altering species interaction patterns will work synergistically with genetic and epidemiological research. Studies have increasingly demonstrated the importance of climatic and anthropogenic change on parasite and vector life cycles, but the ecology of the hosts is generally understudied. For example, mathematical modeling suggests that group size plays an important role in disease transmission dynamics and infection rates (Caillaud et al., 2013; Nunn et al., 2015), though these questions have generally not been explored for multi-host systems. Similarly, more attention is needed on how the timing of activity patterns of hosts, vectors, and parasites influences infection risk within and across species, including in relation to drug performance (Owolabi et al., 2021). Multi-host mathematical models can be a key tool in understanding the factors driving host sharing and infection distributions, especially with changes to local environments through climatic change and human influence. Better feedback or integration across disciplines, including mathematics, social sciences, and different fields of biology, will be an important step, and builds on similar interdisciplinarity in other areas of evolutionary medicine.

Disease burdens, as well as resources, are not equally distributed between or within countries. Within-country capacity building is particularly important for monitoring primate and zoonotic malaria. It is especially important to provide that capacity building for new genomic technologies is facilitating noninvasive sampling and field-based sequencing. Improving the ability of rapid diagnostic tests or designing capture methods to differentiate between closely related parasite species from different hosts – such as *P. vivax* versus *P. simium* or *P. falciparum* from other *Laverania* species – will be an important next step to stop human-NHP transmission pathways.

Recent studies have produced hundreds to thousands of *Plasmodium* genome sequences from around the world, particularly *P. falciparum* samples from Africa and Asia, and to a lesser extent *P. vivax* (Hupalo et al., 2016; Pearson et al., 2016; Amambua-Ngwa et al., 2019). Robust population-genetic methods designed to analyze the plethora of parasite samples are limited, however. Over the past few years, newer work has developed identity-by-descent and linkage-based summaries of genetic variation in *Plasmodium* species more suited to study recent population history (Schaffner et al., 2018; Taylor et al., 2020; de Oliveira et al., 2020; Neafsey et al., 2021). Despite these advances, a major challenge remains: the expected neutral genetic diversity in malaria parasites is unclear, limiting population-genetic inference. *Plasmodium* parasites undergo a complex life cycle through host and vector stages. Multiple steps involve processes expected to dramatically influence genetic variation, such as bottlenecks with transmission, rapid asexual population growth in primate hosts, and sexual recombination in vectors. The combination of these stages makes genetic variation difficult to predict or fit to common models, such as Wright–Fisher (Parobek et al., 2016; Chang and Hartl, 2015; Chang et al., 2013). In turn, common outlier approaches to detect loci of importance (e.g., for drug resistance or adaptation to new hosts), such as *F*_ST_ scans, are difficult to interpret and may produce spurious results.

A handful of studies have begun to address the challenge of understanding genetic variation by using simulation approaches, making substantial progress linking epidemiological and population-genetic models (Chang and Hartl, 2015; Chang et al., 2013; Hendry et al., 2020; Watson et al., 2021). These studies represent an important first step, but progress is needed to incorporate realistic population and genome sizes in computationally tractable ways. Beyond model building for an intuition of the population genetics of malaria infection dynamics, simulation-based studies also open the door for statistical inference of complex processes (Beaumont, 2010). With this model-based inference, we can more confidently differentiate between transmission networks or characterize regions of parasite genomes with patterns outside those expected from demography alone.

As genomic data for a wider variety of parasites becomes available, inference from population-genetic methods will instead be limited by our ecological and biological understanding of the systems. For example, more accurate estimates of mutation rates, the complexities of generation times, recombination rates, mosquito birth and death rates, and coinfection probability are needed to parameterize the models. An important next step will be to explore the phenotypic consequences and origins of the observed genetic variants hypothesized to play a role in host switching or transmission. To achieve this goal, functional tests are needed, together with population-genetic inference of adaptive versus neutral processes. Experimental studies have identified key pathways in *P. falciparum* that likely played a role in host switching from its putative ancestors in gorillas (Proto et al., 2019; Galaway et al., 2019). *P. vivax*, however, is not amenable to long-term lab culture, so functional experiments have been limited and population-genetic data will play a central role (Luo et al., 2015; Udomsangpetch et al., 2008; Noulin et al., 2013). Combining genomic information with temporally and geographically fine-scale epidemiological and ecological data will better enable scientists to link human activity to vector-borne disease prevalence and aid in predicting and prioritizing emerging zoonoses. Archived or historical samples from humans and primates are rich resources that are only beginning to be utilized (van Dorp et al., 2020). Connecting diverse information types will need to move beyond simple correlations to model-based inference and a deeper understanding of the mechanisms behind human-environment-disease interactions.

## Additional files

Supplementary file 1. Nonhuman primate (NHP) malaria occurrence database. Reported outcomes of tests for *Plasmodium* species sampled from NHPs. Sample location, method of sampling, sample size, and parasite and host species are included with original reference. Builds upon the occurrence database of Faust and Dobson, 2015. Further details are provided in the main text and Section 2.

Supplementary file 2. Zoonotic malaria occurrence database. Reported outcomes of tests for nonhuman primate (NHP) *Plasmodium* parasites sampled in humans. Sample location, method of sampling, sample size, and parasite species re included with original reference. Further details are provided in main text and Section 2.
